# A Mother’s Gift: Congenital Transmission of *Trypanosoma* and *Leishmania* Species

**DOI:** 10.1371/journal.ppat.1005302

**Published:** 2016-01-28

**Authors:** Tara Grinnage-Pulley, Benjamin Scott, Christine A. Petersen

**Affiliations:** Department of Epidemiology, College of Public Health, University of Iowa, Iowa City, Iowa, United States of America; University of Wisconsin Medical School, UNITED STATES

## Are Kinetoplastids Transmitted Vertically?

Kinetoplastids *Leishmania infantum* and *Trypanosoma cruzi*, the causative agents of zoonotic visceral leishmaniasis (ZVL) and Chagas’ disease (CD), respectively, can both be transmitted vertically in utero from mother to infant [1]. Congenital infection can be either asymptomatic infection or overt, symptomatic infection [2].

Dogs are a primary domestic reservoir associated with human CD in certain endemic areas and with human ZVL where *L*. *infantum* infection predominates. CD, or American Trypanosomiasis, is endemic in 21 countries within the Americas, where classical transmission is via reduviid bugs. Dogs can present with clinical CD. Although rarer, vertical transmission of CD has been reported in dogs. *L*. *infantum (*syn. *L*. *chagasi*) is one of the *Leishmania* species causing visceral leishmaniasis (VL). *L*. *donovani*, another causative agent of VL and predominantly a human pathogen, has not demonstrated vertical transmission, but this is an understudied area. *L*. *infantum* is endemic in the Americas and the Mediterranean region, with primary transmission by sand fly. Although both *Lutzomyia shannoni* and *Lu*. *anthophora*, present in North America, are potential vectors for *Leishmania* spp. [3], sand fly transmission of *L*. *infantum* in North America has not been confirmed. VL is not endemic in North American human populations, but it is endemic in hunting dogs due to vertical transmission of *L*. *infantum* [4,5].

## What Is the Scope of Vertically Transmitted Kinetoplastid Disease and How Is It Detected?

**Fig 1:**
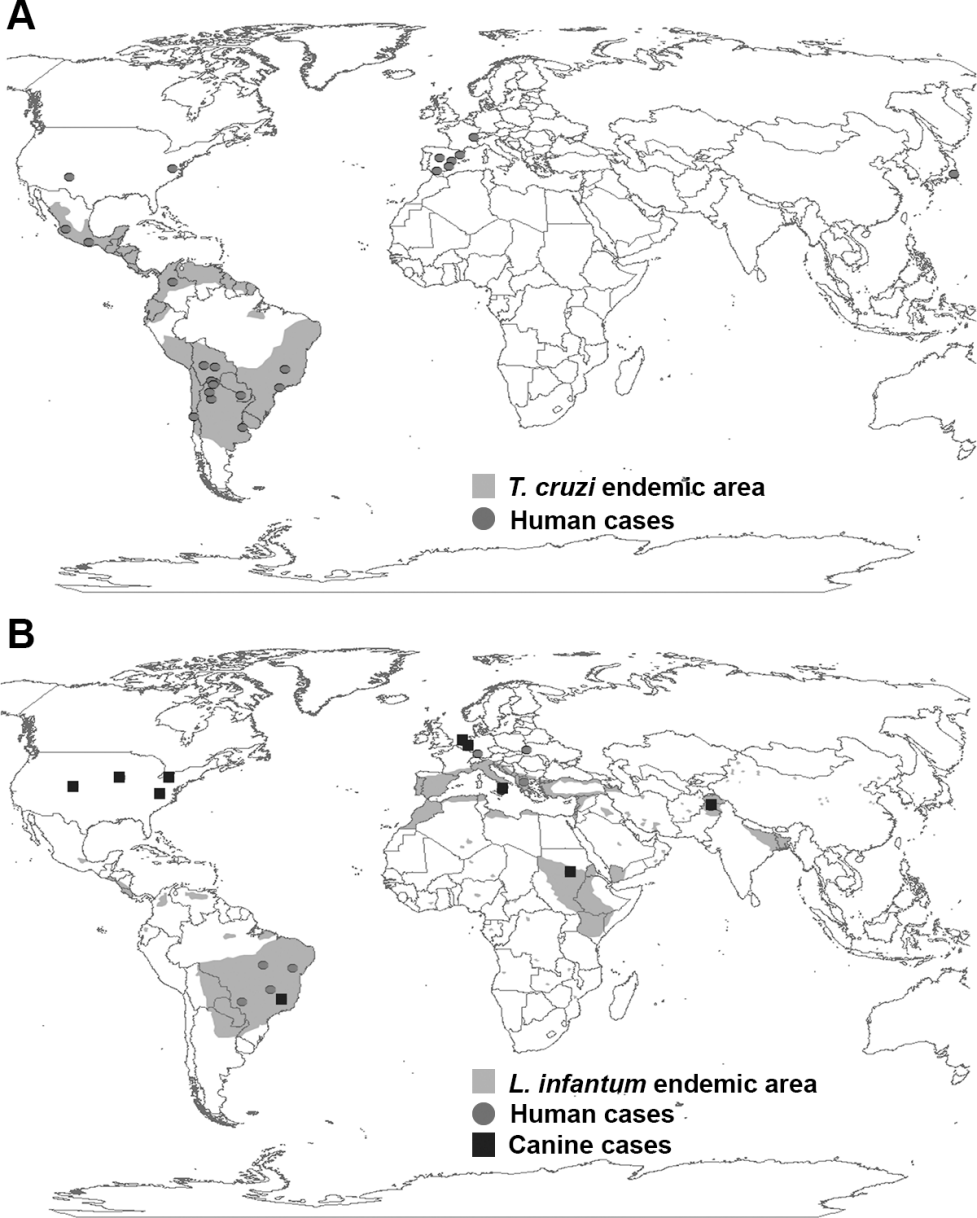
Geographic distribution of *T*. *cruzi* and *L*. *infantum* endemic areas and congenitally transmitted cases as reported in peer-reviewed literature. Endemic regions for (A) *T*. *cruzi* and (B) *L*. *infantum* are shaded. Gray dots represent at least one report of human congenital infection with (A) *T*. *cruzi* or (B) *L*. *infantum*. Black squares represent at least one report of (B) canine congenital infection with *L*. *infantum*. Maps created with ArcMap, Version 10.2 (Esri). See S1 File for case and location references.

Global travel and migration has brought people and animals with CD and VL to non-endemic regions (Fig 1). CD is estimated to affect 6–7 million people annually [6,7]. There are an estimated 2 million women of child-bearing age in the Americas at risk of congenitally transmitting CD [8]. Seroprevalence in pregnant women ranges from <1% to 64% based on location [5], with estimates of vertical transmission rates varying geographically from 1% to 12% [8,9]. The average transmission rate is 5% (Fig 1A) [2,9]. In Latin America, this results in an estimated 8,700 to 15,000 annual congenital cases of CD [2,5,6,10]. Interestingly, there have been several cases of vertical transmission occurring in familial clusters [11,12]. Fourteen cases of *T*. *cruzi* transmission in multiple pregnancies resulted in families with congenitally infected siblings [13]. Four cases had transmission across two generations, from grandmother to mother to child [13]. Underlying reasons for the familial clustering, beyond lack of diagnosis, were unknown.

These case reports, along with a survey of United States obstetrician-gynecologists with 70% of respondents having “very limited” knowledge of CD, highlight the need for awareness of congenital CD for better diagnosis and to prevent additional occurrence of congenital CD from any infected mother [11–14]. Diagnosis of congenital CD is complicated. It requires confirmation of chronic maternal infection through serologic, molecular (PCR-based), or rapid (immunochromogenic, immunodot, etc.) diagnostics [1,15–19]. Serologic tests include enzyme immunoassay (EIA), enzyme-linked immunosorbent assay (ELISA), or indirect immunofluorescent assay (IFA), although cross reaction with *Leishmania* spp. is a potential problem, diminishing the reliability of antibody-based assays as first-line diagnostics [20]. Depending on diagnostic used, confirmatory tests may be necessary [1,15,20]. Use of serology to diagnose congenital infection in infants born to infected mothers is complicated by the presence of maternal antibodies until about 8 months of age [1,21]. Serologic testing is recommended at 1, 6, and 12 months of age [1,21]. If possible, testing should be performed in conjunction with microscopic detection of live parasites in umbilical cord blood or the infant’s peripheral blood, as parasitemia can be detected during the acute phase of infection [1]. Giemsa stain of blood smears or buffy coat can be performed any time between birth and 6 months of age to diagnose congenital CD but is most effective in the first month to isolate exposure of the infant to maternal sources [1,2]. Congenital infections are confirmed with positive identification of parasites prior to 6 months of age and/or seropositivity at 10 months of age or older [21]. Non-infected children should be seronegative after 10 months and have no detectable parasites via blood smear microscopy [1]. PCR of umbilical cord blood and infant peripheral blood has been shown to detect infection a few weeks after birth and can be beneficial in diagnosis of cases with low parasite transmission [22,23].

There are an estimated 0.2–0.4 million cases of VL each year, causing 20,000–40,000 deaths annually [24]. However, unlike CD, the number of case reports of vertical *L*. *infantum* transmission in humans are limited (Fig 1B, gray dots) [8]. The lack of human congenital cases is attributed to the lack of awareness of VL by health care providers, particularly in non-endemic settings, and/or availability of treatment of mothers for ZVL during pregnancy to reduce the maternal parasite load [25,26]. In contrast, treatment for CD during pregnancy is not advised due to teratogenic potential of available drugs [1].

The majority of reports of ZVL vertical transmission are from the reservoir host, dogs. Until recently, maintenance of canine disease was thought to be due only to vector-borne transmission in dogs living in or traveling to endemic areas. In dogs, natural vertical *L*. *infantum* transmission is increasingly recognized to occur in both endemic and non-endemic countries (Fig 1B, black squares) [27–29]. Canine ZVL seroprevalence rates range from 4% to 57% based on location [5]. Estimates of vertical transmission rates are difficult to determine due to the lack of a specific marker for vertical transmission within the canine population, particularly in endemic areas where vector-borne transmission occurs.

In North America, where VL is endemic in hunting dogs, seroprevalence ranges from 8.8% to 9.8% [4]. Vertical transmission was induced experimentally in a beagle in 2005 [30] and suspected in two sibling dogs in 2008 [31]. Natural vertical transmission was confirmed in a 2011 report of infected pups [27,32]. Similarly to human congenital infections, there are multiple instances of familial clustering [33] and multigenerational spread. This may highlight the possibility of genetic predisposition and has been validated in both dogs and humans [34]. Canine cases have been diagnosed by serology (ELISA, indirect fluorescent-antibody test [IFAT], or others), molecular tests, (quantitative polymerase chain reaction [qPCR]), or rapid immunochromogenic tests such as the Dual Path Platform canine visceral leishmaniasis test (DPP CVL) [33,35–37]. qPCR on maternal blood or on blood or tissues from pups can be used to detect infection and is available in conjunction with other testing methods [4,27,33]. Culling of positive dogs is used by some breeding kennels once vertical transmission has been detected and is used to control vector-borne disease and, presumptively, vertical transmission in endemic areas [38]. Due to conflicts between test-and-cull programs and test-and-treat/sterilize or other potential campaigns to halt vertical transmission within dogs in endemic areas, there is a dramatic disparity between international efforts to address congenital CD in humans and vertical VL in the reservoir host for human ZVL, dogs.

## What Are Signs and Symptoms of Congenitally Transmitted CD or VL?

The majority of *T*. *cruzi*-infected women acquire *T*. *cruzi* through vector transmission. Vertically transmitting mothers are usually in the asymptomatic phase when pregnant, and most pregnancies are without complication [13]. The majority of infected infants have asymptomatic CD, but a proportion will develop chronic disease with cardiac and/or gastrointestinal symptoms years later [2,21]. In cases of overt congenital CD, symptoms are present at birth or appear days to weeks later [1,21]. Symptoms are nonspecific with low birth weight (in up to 40% of births), prematurity, reduced growth rates, hepatomegaly, splenomegaly, and jaundice [21]. Respiratory distress frequently occurs secondary to prematurity or due to parasite-induced pneumonitis [21]. Severe CD occasionally presents with meningoencephalitis with or without microencephaly, acute myocarditis with cardiomegaly and arrhythmias, anemia, and thrombocytopenia [21]. Rarely chorioretinitis, opaque vitreous body, megaesophagous, and megacolon occurs, with fatality more likely if gastrointestinal symptoms are present. Mortality is approximately 5% and is directly correlated with severity of symptoms [21].

Canine vertical ZVL is progressive, with vertically-infected dogs initially asymptomatic. As signs and symptoms appear, they are at first nonspecific (generalized lymphadenopathy, depression), then more pronounced (serosangineous nasal discharge and dull hair coat) [4,5]. Signs are more severe and pathognomonic for ZVL later in disease: splenomegaly, hepatomegaly, loss of body condition [4,5]. Notably, infected females are more often asymptomatic at breeding, but secondary immunosuppression such as concurrent tick-borne disease or pregnancy alone initiates seroconversion and progressive ZVL [4]. Congenitally infected pups are often asymptomatic and remain so for months to years until secondary immunosuppression occurs [4]. Mortality rates for congenital ZVL in dogs are unknown.

## What Are Maternal Immunologic Risk Factors for Vertical Transmission?

*T*. *cruzi* transmission risk increases with higher maternal parasite load and/or reactivated disease [1,21]. Mothers who transmit *T*. *cruzi* to their infants often have decreased TNF-α and IFN-γ serum cytokine levels, decreased expression of CD54 and HLA-DR on CD14^+^ monocytes, and increased *T*. *cruzi*-specific IgM compared to non-transmitting infected women [21]. Non-transmitting women have higher levels of serum pro-inflammatory cytokines, IL1-β, IL-5, and TNF-α [21]. Maternal coinfection with HIV or malaria increases risk of transmission [1,21]. CD transmission through breast milk has not been confirmed [21,39]. Risk for congenital infection increases with placental trophoblastic parasite load and decreased placental IFN-γ and TNF-α levels. Non-infected infants from infected mothers have 60% higher TNF-R2 receptor levels [21]. It is unclear whether there are specific *T*. *cruzi* factors that promote vertical transmission [2]. Genotypes TcII and TcV, which have different tissue tropisms, are known to be transmitted vertically, but it is unknown if additional genotypes can also cause congenital infection [2].

Leishmaniasis transmission also occurs transplacentally in dogs, but the exact timing is unknown. Pregnant females coinfected with helminths, rickettsial disease, heartworm, and other diseases are at greater risk of transmission due to immunosuppression [5]. Defense against leishmaniasis requires a Th1 response with IL-12 and IFN-γ secretion by CD4^+^ T cells. Presence of these immune factors reduces the risk of transmission from dam to pup [33,40]. In active ZVL, increased IL-10 and blood parasite load is observed along with decreased IFN-γ [40]. As severity increases, CD4^+^ T cell proliferation and IFN-γ levels decrease, indicating a state of T cell exhaustion, while IL-10 rises, [40] increasing maternal transmission risk. In congenitally infected, asymptomatic pups, CD4^+^ T cells retain the ability to proliferate in response to parasite antigens. In response to persistent infection in the face of inflammation, these dogs also have decreased T cell proliferation as disease progresses and symptoms appear [40,41]. Parasite-specific factors for vertical transmission of VL are unknown but may be important for targeting placental interaction.

## What Can Be Done to Reduce the Risk of Vertical Transmission?

CD and ZVL vertical transmission risk increases when mothers have an increased parasite load, are co-infected with other pathogens, or have other co-morbid immunosuppressive disease with subsequent lack of an appropriate immune response to *T*. *cruzi* or *L*. *infantum* infection [1,5,21,33,40]. Detection of maternal infection is critical. If done prior to pregnancy, the mother can seek treatment and may consider delaying pregnancy until treatment is complete. CD cannot be treated during pregnancy, although treatment of pregnant women for ZVL is possible [1,21,25,26]. Dogs positive for ZVL are not recommended for breeding. If a pregnant mother is positive for either CD or VL, testing of the infant is recommended to limit disease manifestations, as vertical transmission has already occurred [1,42]. Informing gynecologists and general practitioners with at-risk (born in endemic area) patients of child-bearing age about CD and ZVL is imperative. This will promote patient education and accurate assessment of congenital infection risk [26,42].

## Supporting Information

S1 File Map methods and references. (DOCX)
