# Supplementary material for: A Mother’s Gift: Congenital Transmission of Trypanosoma and Leishmania Species
Source: PLoS Pathog. 2016 Jan 28;12(1):e1005302. doi: 10.1371/journal.ppat.1005302 (PMC4731145; doi:10.1371/journal.ppat.1005302)
Supplement: S1 File — (DOCX) [file ppat.1005302.s001.docx]

S1 Map methods and references

**Map creation methods**

Maps in Fig 1 were created by importing the World Map shapefile [1] into ArcMap Version 10.2 (Esri) to define country borders then cases were plotted for Chagas disease

[2-22] and visceral leishmaniasis based on review of the literature [23-40]. Endemic regions were drawn based on maps of endemic regions for Chagas disease [41, 42] and visceral leishmaniasis [43].

**References**

1. Sandvik, B, cartographers. World Borders Dataset [shapefile]. 2008 [Accessed August 2015]; Available from: http://www.thematicmapping.org/downloads/world_borders.php

2. Apt W, Zulantay I, Arnello M, Oddó D, González S, Rodríguez J, et al. Congenital infection by Trypanosoma cruzi in an endemic area of Chile: a multidisciplinary study. Trans R Soc Trop Med Hyg. 2013;107(2):98-104. doi: 10.1093/trstmh/trs013. PubMed PMID: 23222947.

3. Apt W, Zulantay I, Solari A, Ortiz S, Oddo D, Corral G, et al. Vertical transmission of Trypanosoma cruzi in the Province of Choapa, IV Region, Chile: Preliminary Report (2005-2008). Biol Res. 2010;43(3):269-74. doi: /S0716-97602010000300002. PubMed PMID: 21249297.

4. Barona-Vilar C, Giménez-Martí MJ, Fraile T, González-Steinbauer C, Parada C, Gil-Brusola A, et al. Prevalence of Trypanosoma cruzi infection in pregnant Latin American women and congenital transmission rate in a non-endemic area: the experience of the Valencian Health Programme (Spain). Epidemiol Infect. 2012;140(10):1896-903. doi: 10.1017/S0950268811002482. PubMed PMID: 22129521.

5. Bern C, Verastegui M, Gilman RH, Lafuente C, Galdos-Cardenas G, Calderon M, et al. Congenital Trypanosoma cruzi transmission in Santa Cruz, Bolivia. Clin Infect Dis. 2009;49(11):1667-74. doi: 10.1086/648070. PubMed PMID: 19877966.

6. Blanco SB, Segura EL, Cura EN, Chuit R, Tulián L, Flores I, et al. Congenital transmission of Trypanosoma cruzi: an operational outline for detecting and treating infected infants in north-western Argentina. Trop Med Int Health. 2000;5(4):293-301. PubMed PMID: 10810029.

7. Brutus L, Castillo H, Bernal C, Salas NA, Schneider D, Santalla JA, et al. Detectable Trypanosoma cruzi parasitemia during pregnancy and delivery as a risk factor for congenital Chagas disease. Am J Trop Med Hyg. 2010;83(5):1044-7. doi: 10.4269/ajtmh.2010.10-0326. PubMed PMID: 21036835; PubMed Central PMCID: PMCPMC2963967.

8. Brutus L, Schneider D, Postigo J, Delgado W, Mollinedo S, Chippaux JP. Evidence of congenital transmission of Trypanosoma cruzi in a vector-free area of Bolivia. Trans R Soc Trop Med Hyg. 2007;101(11):1159-60. doi: 10.1016/j.trstmh.2007.03.015. PubMed PMID: 17499827.

9. Bua J, Volta BJ, Velazquez EB, Ruiz AM, Rissio AM, Cardoni RL. Vertical transmission of Trypanosoma cruzi infection: quantification of parasite burden in mothers and their children by parasite DNA amplification. Trans R Soc Trop Med Hyg. 2012;106(10):623-8. doi: 10.1016/j.trstmh.2012.03.015. PubMed PMID: 22835758.

10. Cardoso EJ, Valdéz GC, Campos AC, de la Luz Sanchez R, Mendoza CR, Hernández AP, et al. Maternal fetal transmission of Trypanosoma cruzi: a problem of public health little studied in Mexico. Exp Parasitol. 2012;131(4):425-32. doi: 10.1016/j.exppara.2012.05.013. PubMed PMID: 22683499.

11. (CDC) CfDCaP. Congenital transmission of Chagas disease - Virginia, 2010. MMWR Morb Mortal Wkly Rep. 2012;61(26):477-9. PubMed PMID: 22763884.

12. Flores-Chávez M, Faez Y, Olalla JM, Cruz I, Gárate T, Rodríguez M, et al. Fatal congenital Chagas' disease in a non-endemic area: a case report. Cases J. 2008;1(1):302. doi: 10.1186/1757-1626-1-302. PubMed PMID: 18992159; PubMed Central PMCID: PMCPMC2585580.

13. Flores-Chavez MD, Merino FJ, Garcia-Bujalance S, Martin-Rabadan P, Merino P, Garcia-Bermejo I, et al. Surveillance of Chagas disease in pregnant women in Madrid, Spain, from 2008 to 2010. Euro Surveill. 2011;16(38). PubMed PMID: 21958533.

14. Hermann E, Truyens C, Alonso-Vega C, Rodriguez P, Berthe A, Torrico F, et al. Congenital transmission of Trypanosoma cruzi is associated with maternal enhanced parasitemia and decreased production of interferon- gamma in response to parasite antigens. J Infect Dis. 2004;189(7):1274-81. doi: 10.1086/382511. PubMed PMID: 15031797.

15. Imai K, Maeda T, Sayama Y, Mikita K, Fujikura Y, Misawa K, et al. Mother-to-child transmission of congenital Chagas disease, Japan. Emerg Infect Dis. 2014;20(1):146-8. doi: 10.3201/eid2001.131071. PubMed PMID: 24378113; PubMed Central PMCID: PMCPMC3884731.

16. Jackson Y, Myers C, Diana A, Marti HP, Wolff H, Chappuis F, et al. Congenital transmission of Chagas disease in Latin American immigrants in Switzerland. Emerg Infect Dis. 2009;15(4):601-3. doi: 10.3201/eid1504.080438. PubMed PMID: 19331743; PubMed Central PMCID: PMCPMC2671437.

17. Luquetti AO, Tavares SB, Siriano LaR, de Oliveira RA, Campos DE, de Morais CA, et al. Congenital transmission of Trypanosoma cruzi in central Brazil. A study of 1,211 individuals born to infected mothers. Mem Inst Oswaldo Cruz. 2015;110(3):369-76. doi: 10.1590/0074-02760140410. PubMed PMID: 25993506; PubMed Central PMCID: PMCPMC4489474.

18. Muñoz J, Coll O, Juncosa T, Vergés M, del Pino M, Fumado V, et al. Prevalence and vertical transmission of Trypanosoma cruzi infection among pregnant Latin American women attending 2 maternity clinics in Barcelona, Spain. Clin Infect Dis. 2009;48(12):1736-40. doi: 10.1086/599223. PubMed PMID: 19438393.

19. Murcia L, Carrilero B, Munoz-Davila MJ, Thomas MC, López MC, Segovia M. Risk factors and primary prevention of congenital Chagas disease in a nonendemic country. Clin Infect Dis. 2013;56(4):496-502. doi: 10.1093/cid/cis910. PubMed PMID: 23097582.

20. Nisida IV, Amato Neto V, Braz LM, Duarte MI, Umezawa ES. A survey of congenital Chagas' disease, carried out at three health institutions in São Paulo City, Brazil. Rev Inst Med Trop Sao Paulo. 1999;41(5):305-11. PubMed PMID: 10602545.

21. Pavia PX, Montilla M, Flórez C, Herrera G, Ospina JM, Manrique F, et al. [The first case of congenital Chagas' disease analyzed by AP-PCR in Colombia]. Biomedica. 2009;29(4):513-22. PubMed PMID: 20440449.

22. Sánchez Negrette O, Mora MC, Basombrío MA. High prevalence of congenital Trypanosoma cruzi infection and family clustering in Salta, Argentina. Pediatrics. 2005;115(6):e668-72. doi: 10.1542/peds.2004-1732. PubMed PMID: 15930194.

23. Boggiatto PM, Gibson-Corley KN, Metz K, Gallup JM, Hostetter JM, Mullin K, et al. Transplacental transmission of Leishmania infantum as a means for continued disease incidence in North America. PLoS Negl Trop Dis. 2011;5(4):e1019. doi: 10.1371/journal.pntd.0001019. PubMed PMID: 21532741; PubMed Central PMCID: PMC3075227.

24. Cabello PH, Lima AM, Azevedo ES, Krieger H. Familial aggregation of Leishmania chagasi infection in northeastern Brazil. Am J Trop Med Hyg. 1995;52(4):364-5. Epub 1995/04/01. PubMed PMID: 7741179.

25. da Silva SM, Ribeiro VM, Ribeiro RR, Tafuri WL, Melo MN, Michalick MS. First report of vertical transmission of Leishmania (Leishmania) infantum in a naturally infected bitch from Brazil. Vet Parasitol. 2009;166(1-2):159-62. Epub 2009/09/08. doi: 10.1016/j.vetpar.2009.08.011. PubMed PMID: 19733439.

26. Diaz-Espineira MM, Slappendel RJ. A case of autochthonous canine leishmaniasis in the Netherlands. Veterinary Quarterly. 1997;19(2):69-71. PubMed PMID: CABI:19972212422.

27. Eltoum IA, Zijlstra EE, Ali MS, Ghalib HW, Satti MM, Eltoum B, et al. Congenital kala-azar and leishmaniasis in the placenta. Am J Trop Med Hyg. 1992;46(1):57-62. PubMed PMID: 1536385.

28. Figueiró-Filho EA, El Beitune P, Queiroz GT, Somensi RS, Morais NO, Dorval ME, et al. Visceral leishmaniasis and pregnancy: analysis of cases reported in a central-western region of Brazil. Arch Gynecol Obstet. 2008;278(1):13-6. doi: 10.1007/s00404-007-0532-0. PubMed PMID: 18087708.

29. Freeman KS, Miller MD, Breitschwerdt EB, Lappin MR. Leishmaniasis in a dog native to Colorado. J Am Vet Med Assoc. 2010;237(11):1288-91. doi: 10.2460/javma.237.11.1288. PubMed PMID: 21118014.

30. Gaskin AA, Schantz P, Jackson J, Birkenheuer A, Tomlinson L, Gramiccia M, et al. Visceral leishmaniasis in a New York foxhound kennel. Journal of Veterinary Internal Medicine. 2002;16(1):34-44. PubMed PMID: WOS:000173430700005.

31. Gibson-Corley KN, Hostetter JM, Hostetter SJ, Mullin K, Ramer-Tait AE, Boggiatto PM, et al. Disseminated Leishmania infantum infection in two sibling foxhounds due to possible vertical transmission. Can Vet J. 2008;49(10):1005-8. PubMed PMID: 19119370; PubMed Central PMCID: PMC2553493.

32. Maciel DB, Silva TA, Gomes LI, de Oliveira E, Tibúrcio MG, de Oliveira RF, et al. Infection with Leishmania (Leishmania) infantum of 0 to 18-Month-old children living in a visceral leishmaniasis-endemic area in Brazil. Am J Trop Med Hyg. 2014;91(2):329-35. doi: 10.4269/ajtmh.13-0418. PubMed PMID: 24935952; PubMed Central PMCID: PMCPMC4125257.

33. Mancianti F, Sozzi S. Isolation of Leishmania from a newborn puppy. Transactions of the Royal Society of Tropical Medicine and Hygiene. 1995;89(4):402. PubMed PMID: CABI:19950809951.

34. Meinecke CK, Schottelius J, Oskam L, Fleischer B. Congenital transmission of visceral leishmaniasis (Kala Azar) from an asymptomatic mother to her child. Pediatrics. 1999;104(5):e65. Epub 1999/11/05. PubMed PMID: 10545591.

35. Mescouto-Borges MR, Maués É, Costa DL, Pranchevicius MC, Romero GA. Congenitally transmitted visceral leishmaniasis: report of two Brazilian human cases. Braz J Infect Dis. 2013;17(2):263-6. doi: 10.1016/j.bjid.2012.10.017. PubMed PMID: 23453409.

36. Naucke TJ, Lorentz S. First report of venereal and vertical transmission of canine leishmaniosis from naturally infected dogs in Germany. Parasites and Vectors. 2012;5(67):(1 April 2012). PubMed PMID: CABI:20123134548.

37. Papageorgiou T, Pana Z, Tragiannidis A, Tsotoulidou V, Pratsiou E, Tzouvelekis G, et al. The first case of congenital leishmaniasis in a female infant in Greece. J Paediatr Child Health. 2010;46(10):611-2. doi: 10.1111/j.1440-1754.2010.01862.x. PubMed PMID: 20958824.

38. Patel JM, Rosypal AC, Zimmerman KL, Monroe WE, Sriranganathan N, Zajac AM, et al. Isolation, mouse pathogenicity, and genotyping of Trypanosoma cruzi from an English Cocker Spaniel from Virginia, USA. Veterinary Parasitology. 2012;187(3-4):394-8. PubMed PMID: WOS:000307323700006.

39. Rab MA, Mahmood MT, Bux D, Hassan M, Hassan K. Visceral leishmaniasis in a six month old child: is congenital transmission of disease possible? J Pak Med Assoc. 1991;41(8):191-3. PubMed PMID: 1942482.

40. Zinchuk A, Nadraga A. Congenital visceral leishmaniasis in Ukraine: case report. Annals of Tropical Paediatrics. 2010;30(2):161-4. PubMed PMID: WOS:000278169500011.

41. Control of Chagas disease. Report of a WHO Expert Committee. World Health Organ Tech Rep Ser. 1991;811:1-95. PubMed PMID: 1746164.

42. Coura JR, Dias JC. Epidemiology, control and surveillance of Chagas disease: 100 years after its discovery. Mem Inst Oswaldo Cruz. 2009;104 Suppl 1:31-40. PubMed PMID: 19753455.

43. WHO/NTD/IDM HIV/AIDS TaMHWHO, cartographer. Essential leishmaniasis maps: Geographical distribution of visceral leishmaniasis in the Old and New World [map]. 2010 [Accessed August 2015]; Available from: <http://www.who.int/leishmaniasis/leishmaniasis_maps/en/>
